# Two Cases of Methemoglobinemia Induced by the Exposure to Nitrobenzene and Aniline

**DOI:** 10.1186/2052-4374-25-31

**Published:** 2013-11-01

**Authors:** Chang Hwan Lee, Soo Hyeon Kim, Do Hyung Kwon, Keun Ho Jang, Yong Hoon Chung, Jai Dong Moon

**Affiliations:** 1 Department of Occupational and Environmental Medicine, Chonnam National University Hwasun Hospital, Hwasun, Korea; 2 Department of Emergency Medicine, Chonnam National University Hospital, Gwangju, Korea

**Keywords:** Nitrobenzene, Aniline, Methemoglobinemia

## Abstract

**Objective:**

To report two cases of methemoglobinemia induced by inhaled nitrobenzene and dermally absorbed aniline.

**Methods:**

We have evaluated a 37-year-old male worker exposed to nitrobenzene by inhalation while conducting maintenance job of mononitrobenzene pump and a 25-year-old male worker exposed dermally to aniline while unloading.

**Results:**

The first case is a 37-year-old male exposed to nitrobenzene. His blood methemoglobin concentration level was initially 19.8%, and chest X-ray was normal. After oxygen therapy, the blood methemoglobin concentration level decreased to 2.1%, and the symptoms were alleviated. The second case is a 25-year-old male exposed dermally to aniline. His chest X-ray was normal, but blood methemoglobin concentration level reached maximally 46.8%. He was treated with methylene blue due to relatively high blood methemoglobin level. Gradually after the treatment, his methemoglobin concentration level was normalized to 0.8% and simultaneously symptoms were resolved.

**Conclusions:**

After the thorough exposure investigations and medical evaluations, we have concluded that these cases were methemoglobinemia induced by occupational exposure to nitrobenzene and aniline. We suggest that businesses which handle methemoglobinemia-causing substances control the engineering process strictly, implement periodic screening, and establish emergency patient management system.

## Background

Methemoglobin (MetHb) is a modified form of normal hemoglobin where Fe2+ (ferrous ion) is oxidized into Fe3+ (ferric ion). MetHb cannot bind with oxygen, and hence it cannot carry oxygen. The human body can tolerate a very small amount (less than 1%) of MetHb, but a higher level is likely to cause methemoglobinemia [1-4].

Methemoglobinemia can be both inherited and acquired. Methemoglobinemia is commonly caused by exposure to medical substances, such as benzocaine and dapsone, that oxidize hemoglobin to MetHb; exhaust fumes from internal combustion engines; herbicides and pesticides; and chemicals, such as nitrobenzene and aniline [5-11]. Nitrobenzene and aniline are typical aromatic nitro compounds and aromatic amino compounds that cause methemoglobinemia. Nitrobenzene is mostly used in the synthesis of aniline and in the production of benzidine, quinolone, and azobenzene [12]. Nitrobenzene is a pale yellow liquid, which has an almond-like odor at room temperature. The most common paths of occupational exposure to nitrobenzene are inhalation and absorption through the skin [12-15]. The reduction of nitrobenzene to aniline occurs once nitrobenzene is metabolized within the body, and this process oxidizes the hemoglobin in the blood into MetHb, which then causes methemoglobinemia [1,14].

Aniline is a prototypical aromatic amine in a pale yellow liquid form with an unpleasant odor of rotten fish. It is used in the production of dyes, rubber processing chemicals, and antioxidants [16]. The most common occupational exposure to aniline includes inhalation, oral ingestion, and absorption through the skin. Hydroxylamine compounds, such as phenylhydroxylamine, are known to cause methemoglobinemia when they begin oxidation [17-19].

In this paper, we report two cases of methemoglobinemia that occurred in chemical plants following exposure to nitrobenzene and aniline through inhalation and absorption through the skin, as well as a review of the literature related to occupational methemoglobinemia.

## Case presentation

### Case 1

#### Patient

37-year-old male.

#### Chief complaint

Dyspnea, cyanoderma.

#### Past medical history and/or family history

Nothing significant.

#### History of cigarette smoking and alcohol consumption

Nonsmoker, rarely drinks.

#### Occupational history

**Figure 1:**
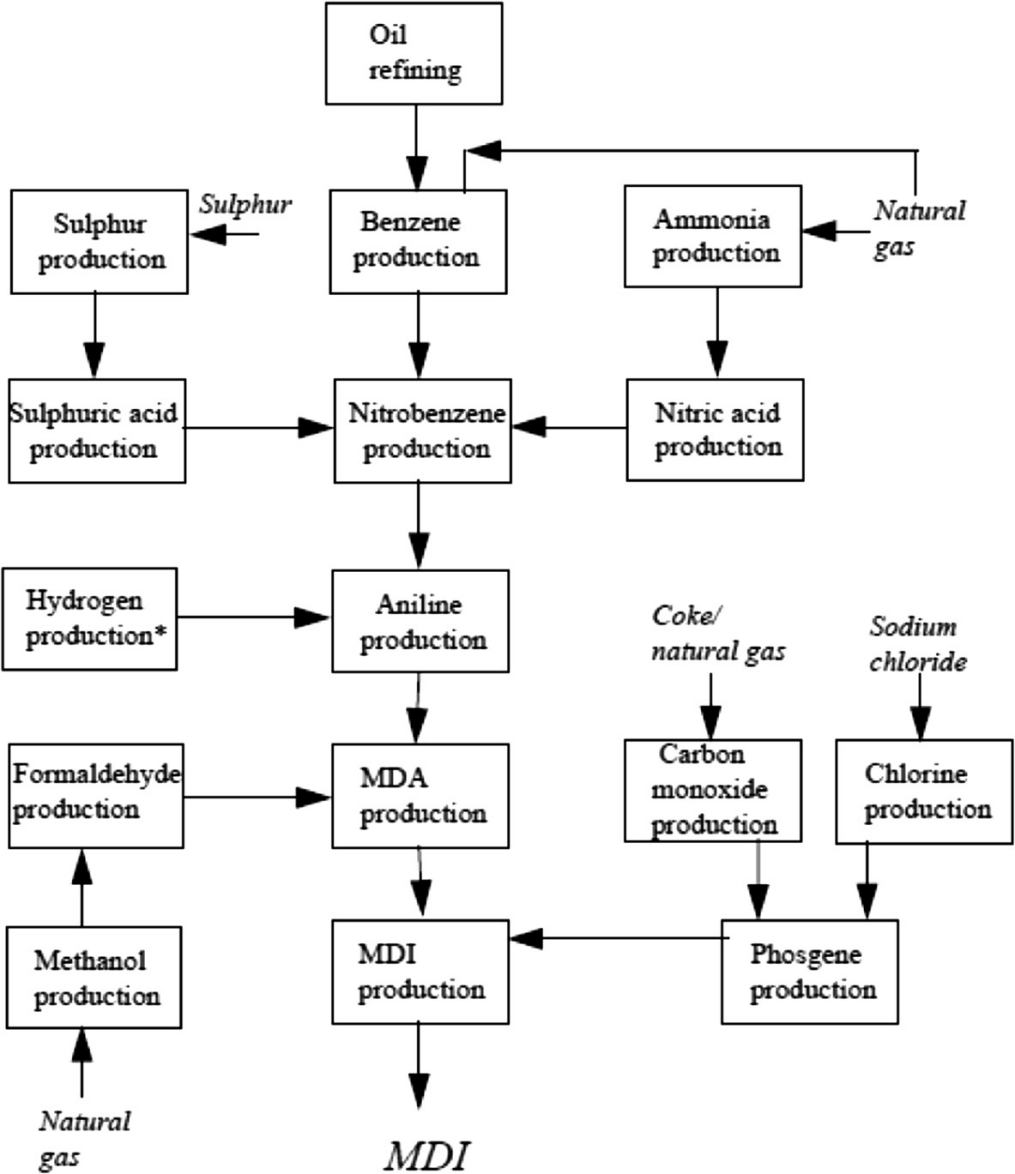
Schematic diagram showing the principal operations leading to the production of MDI.

The patient works in methylene diphenyl diisocyanate (MDI) production (Figure 1). Prior to experiencing symptoms, the patient had been cleaning a suction strainer of a mononitrobenzene pump for six to seven hours. The process of cleaning a suction strainer starts from opening the cover of the strainer, taking out the screen that is inside, removing impurities and foreign substances, and then reassembling the strainer. It is likely that the patient was exposed to mononitrobenzene through inhalation during this process.

#### History of present illness

The patient displayed no symptoms while cleaning the suction strainer of the mononitrobenzene pump, but he experienced dyspnea and cyanoderma shortly after he returned home after work. He was immediately taken to the local hospital. As he showed no improvement after emergency treatment, including oxygen inhalation therapy, he was transferred to the emergency room after consulting the Department of Occupational & Environmental Medicine of an affiliated university hospital in accordance with the emergency patient management guidelines of the workplace.

#### Physical findings

At the time of transfer, his blood pressure was 110/70 mmHg, his pulse rate was 88 beats per minute, his respiratory rate was 24 breaths per minute, and his body temperature was 36.8°C. Although acutely ill, there was no sign of severe cyanoderma anywhere on his body. There were no findings of jaundice in the sclera or conjunctiva pallor according to a head and neck examination. Heart sounds were regular, with no abnormal murmurs. Respiratory sounds were also normal. There were no significant symptoms found in the abdominal region.

#### Laboratory findings

A peripheral blood examination revealed the following: hemoglobin level 15.3 g/dl, hematocrit 43.6%, white blood cells 7,500/mm^3^, and platelet count 188,000/mm^3^. According to a biochemical examination, blood urea nitrogen (BUN) was 14.7 mg/dL, creatinine was 1.0 mg/dL, asparate aminotransferase (AST) was 19 IU/L, and alanine aminotransferase (ALT) was 18 IU/L. Urine test results were normal. An arterial blood gas examination revealed a pH of 7.41, PCO_2_ of 35.6 mmHg, PO_2_ of 161 mmHg, HCO_3_^−^ of 21.9 mmol/L, O_2_ saturation of 97.8%, O_2_Hb (oxidized hemoglobin) of 78.6%, and MetHb of 19.8%.

#### Electrocardiogram findings

An electrocardiogram showed sinus rhythm, but the patient’s heart rate was 60 beats per minute, which was a possible sign of sinus bradycardia.

#### Medical imaging findings

There were no significant findings on plain chest radiography and chest computed tomography.

#### Clinical course

The patient was diagnosed with methemoglobinemia according to the medical test results and his past exposure history to mononitrobenzene. As his MetHb levels were relatively low, he was given simple O_2_ inhalation treatment through a mask at 10 L per minute. Two hours after the treatment, his MetHb was reduced to 12.7%, and his symptoms were alleviated. On his second day of hospitalization, his pH was 7.41, and PCO_2_ was 39.2 mmHg, PO_2_ was 86.3 mmHg, HCO_3_^−^ was 24.5 mmol/L, O_2_ saturation was 95.6%, and MetHb was 2.1% according to an arterial blood gas examination (Table 1). The symptoms of methemoglobinemia, including dyspnea and cyanoderma, disappeared, and he was discharged from the hospital at 1 pm on the same day.

**Table 1:** Progress of blood gas analysis and methemoglobin concentration of case 1

	**Initial**	Progress^*^	
		**2 hours later**	**1 day later**
pH	7.41	7.45	7.41
PCO₂ (mmHg)	35.6	35.2	39.2
PO₂ (mmHg)	161	155	86.3
HCO₃^−^ (mmol/L)	21.9	24.3	24.5
O₂ saturation (%)	97.8	98.1	95.6
Methemoglobin (%)	19.8	12.7	2.1

^*^The patient was treated with O₂ inhalation via mask(10 L/min).

### Case 2

#### Patient

25-year-old male

#### Chief complaint

Signs of cyanoderma appeared one hour and thirty minutes after exposure to aniline.

#### Past medical history and/or family history

Nothing significant.

#### History of cigarette smoking and alcohol consumption

Smoker of 2.5 pack-year, occasionally consumes alcohol

#### Occupational history

The patient works in the MDI production field and is in charge of loading and unloading aniline from bulk trucks. He was exposed to aniline while removing a connector from the tank of a lorry. Approximately 200 cc of aniline that remained in the vent line spattered on his face and his upper body.

#### History of present illness

Following the exposure to the aniline, the patient was taken to a local hospital for emergency treatment and observation. He did not show any immediate symptoms other than pain caused by a burn around the exposed area. However, after O_2_ inhalation treatment (5 L/min via an O_2_ mask), he showed cyanoderma on his entire body, including his face. An arterial blood gas examination revealed O_2_ saturation was reduced from 91% to 89%. The MetHb reading was 26.3%. The patient was transferred to the emergency room after consulting the Department of Occupational & Environmental Medicine of an affiliated university hospital in accordance with the emergency patient management guidelines of the workplace.

#### Physical findings

**Figure 2:**
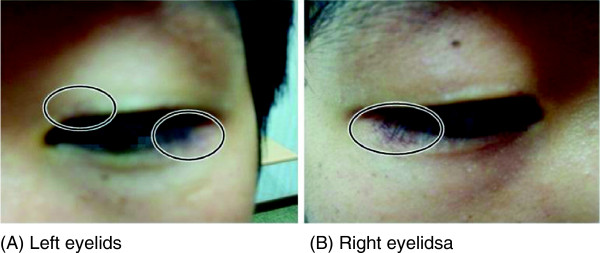
**Skin irritation of upper and lower eyelids of both sides. (A)** Left eyelids. **(B)** Right eyelids.

**Figure 3:**
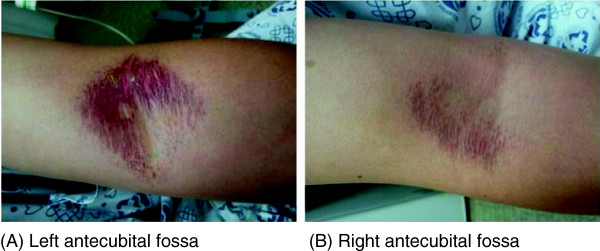
**Skin lesions of antecubital fossa of both arms. (A)** Left antecubital fossa. **(B)** Right antecubital fossa.

At the time of transfer, his blood pressure was 130/80 mmHg, his pulse rate was 80 beats per minute, his respiratory rate was 20 breaths per minute, and his body temperature was 36.0°C. He was acutely ill and showed signs of cyanoderma on his entire body. A head and neck examination showed no findings of jaundice in the sclera or conjunctiva pallor. However, both eyelids showed signs of rubefaction (Figure 2). Heart sounds were regular, with no abnormal murmurs. Respiratory sounds were also normal, but the left side of his chest was red. There were no significant symptoms found in the abdominal region. Redness and blisters were observed in the concave area of both sides of his elbows (Figure 3).

#### Laboratory findings

A peripheral blood examination showed the following: hemoglobin level 14.4 g/dl, hematocrit 41.7%, white blood cells 6,800/mm^3^, and platelet count 218,000/mm^3^. According to a biochemical examination, his BUN was 13.4 mg/dL, creatinine was 0.6 mg/dL, AST was 19 IU/L, and ALT was 18 IU/L. Urine test results were normal. According to the arterial blood gas examination, his pH was 7.41, PCO_2_ was 37.4 mmHg, PO_2_ was 185 mmHg, HCO_3_^−^ was 23.0 mmol/L, O_2_ saturation was 98.3%, O_2_Hb (oxidized hemoglobin) was 51.9%, and MetHb was 46.8%.

#### Electrocardiogram findings

An electrocardiogram showed a sinus rhythm and a regular heart rate (65 beats per minute).

#### Medical imaging findings

There were no significant findings on plain chest radiography.

#### Clinical course

Based on the patient’s medical test results, MetHb level, and exposure history to aniline, the patient was diagnosed with methemoglobinemia. He was given O_2_ inhalation treatment (10 L/min via a mask) and 7 ml of 2% methylene blue diluted in 100 ml of saline solution. His MetHb was 25.3%, 19.3%, 9.9%, and 6.6% at one, two, four, and six hours, respectively after the treatment. According to an arterial blood gas examination on the second day of his hospitalization, his pH was 7.34, PCO_2_ was 47.2 mmHg, PO_2_ was 237 mmHg, HCO_3_^−^ was 25.0 mmol/L, O_2_ saturation was 99.0%, and MetHb was 2.4%. The symptoms caused by the methemoglobinemia, such as cyanoderma, gradually improved. An ointment was applied to skin lesions (primary burns and contact dermatitis) caused by aniline. Eye drops were applied for cornea inflammation. According to an arterial blood gas examination on his fourth day of hospitalization, his pH was 7.46. PCO_2_ was 34.1 mmHg, PO_2_ was 119 mmHg, HCO_3_^−^ was 24.2 mmol/L, O_2_ saturation was 97.2%, and MetHb was 0.8% (Table 2). The level of MetHb returned to normal. The symptoms of methemoglobinemia were gradually alleviated, and he was discharged from the hospital at 2 pm on the seventh day of hospitalization.

**Table 2:** Progress of blood gas analysis and methemoglobin concentration of case 2

	Initial^*^	**Progress**^**†**^						
		**1 hour later**	**2 hours later**	**4 hours later**	**6 hours later**	**1 day later**	**2 days later**	**3 days later**
pH	7.41	7.40	7.40	7.36	7.34	7.34	7.42	7.46
PCO₂ (mmHg)	37.4	41.3	38.1	43.5	45.0	47.2	39,1	34.1
PO₂ (mmHg)	185	221	200.9	261.1	345	237	114	119
HCO₃^−^ (mmol/L)	23.0	23.5	23.1	24.2	23.6	25.0	25.0	24.2
O₂ saturation (%)	98.3	98.5	98.6	99.0	99.2	99.0	98.3	97.2
Methemoglobin (%)	46.8	25.3	19.3	9.9	6.6	2.4	1.5	0.8

^*^The MetHb at the local hospital was 26.3%.

^†^The patient was initially treated with injection of 2% methylene blue and O₂ inhalation.

## Conclusions

As mentioned above, the majority of acquired methemoglobinemia occurs through nonoccupational exposure. Several cases of occupational exposure have been reported during repair and maintenance in production of aniline, medical substances, pesticides, and rubber. Occupational exposure has also occurred during transportation and disposal of substances that cause methemoglobinemia [1,5]. In addition to being uncommon, such exposure is rarely fatal.

Hemoglobin is oxidized into MetHb via three main mechanisms, depending on the source of exposure. Direct oxidization of hemoglobin occurs in response to exposure to specific substances, such as copper (II) sulfate and hexavalent chromates. It relies on the redox potential of the substance relative to hemoglobin. Indirect oxidation of hemoglobin, for example, by nitrites and phenylenediamine, relies on a co-oxidation mechanism. Lastly, there are cases where the substance itself is not sufficient to create MetHb. In such cases, MetHb is created following exposure to specific metabolic activities in the body and subsequent biochemical changes. The occupational exposure cases reported herein where the workers were exposed to aniline and nitrobenzene fall into this category [1].

Occupational exposure to nitrobenzene occurs via inhalation and absorption through the skin. Once it is absorbed, nitrobenzene is metabolized into nitrosobenzene, which is toxic and causes methemoglobinemia [1,14,20]. According to international and domestic studies on nitrobenzene addiction, most exposure takes place via ingestion and absorption through the skin [13-15,21-23].

In the first case, the worker developed symptoms after cleaning a suction strainer of a mononitrobenzene pump for six or seven hours, but he showed no signs of exposure through ingestion or absorption through the skin. The time-weighted average (TWA) of nitrobenzene exposure regulation in Korea is 1 ppm. Markers of nitrobenzene exposure include urinary p-nitrophenol and the aniline concentration level in the blood, but these are nonspecific markers [14,20,24]. One limitation of the current study was the absence of a direct evaluation of the work environment. However, because the worker had no prior drug history or exposure to other chemicals that could have caused methemoglobinemia, his exposure to nitrobenzene during the work process was clear. In addition, as MetHb is often used as a biomarker of nitrobenzene exposure, his methemoglobinemia was diagnosed to be caused by exposure to nitrobenzene.

It is known that occupational exposure to aniline can occur via inhalation through respiratory organs, oral ingestion, and absorption through the skin and that aniline causes methemoglobinemia following oxidation via phenyl hydroxylamine, a metabolite [1,14,17-19]. According to prior cases of aniline exposure, the majority of cases involved oral ingestion and inhalation through the respiratory organs [17-19].

In the second case, the worker was removing a connector from the tank of an aniline lorry when approximately 200 cc of aniline remaining in the vent line spattered on his face and his upper body. Although the possibility of inhalation cannot be ignored, it can be assumed that the major exposure to aniline was absorption through the skin. The TWA of aniline exposure regulation in Korea is 2 ppm, and urinary p-aminophenol is used as a biomarker [19,24]. The worker in this case had no prior intake history of drugs that may have caused the methemoglobinemia, and his methemoglobinemia was diagnosed as due to exposure to aniline.

The diagnosis of methemoglobinemia is based on the results of an arterial blood gas examination and measurements of the concentration of MetHb in the blood [1]. The symptoms of methemoglobinemia differ depending on the concentration level of MetHb in the blood, with 0–20% potentially producing cyanoderma; 20–40% potentially producing headache, anxiety, vertigo, and a frequent pulse; 40–60% potentially producing clouding of consciousness and respiratory failure; and more than 60% potentially resulting in arrhythmia, convulsion, and even death [1,5].

Discontinued exposure to the substance, oxygen therapy, and intravenous injection of methylene blue are some of the options for methemoglobinemia [1,5,25-27]. Under normal circumstances, humans have less than 1% of MetHb in their bodies. In that case, hemoglobin convert to MetHb and MetHb revert to hemoglobin interactively. MetHb may revert to hemoglobin via a reaction involving nicotinamide dinucleotide hydrogenase (NADH) and nicotinamide-adenine dinucleotide phosphate hydrogenase (NADPH). In living tissue, most MetHb is reduced through NADH-dependent reactions rather than via NADPH-dependent reactions. Methylene blue combats methemoglobinemia by activating NADPH reactions and accelerating the reduction process of MetHb [1,4,5]. Methylene blue treatment is applicable when a patient’s MetHb exceeds 25–30% or when the patient exhibits symptoms of oxygen deficiency, such as dyspnea and alteration of consciousness [1,22].

In the first of the presented cases, oxygen treatment alone improved the patient’s symptoms and led to a decrease in his MetHb because his initial MetHb level was 19.8%. In the second case, methylene blue treatment was required because the patient’s MetHb level was 46.8%, and his symptoms improved after the treatment.

The final clinical progress of both workers was positive. According to other reports of occupational exposure to substances that cause methemoglobinemia, fatal outcomes are rare [1]. However, methemoglobinemia is caused by various substances. If specific exposure paths are not diagnosed and understood, early stages of treatment may not be sufficient or appropriate. In extreme cases, this could lead to death. The worker in Case 2 showed no fatal symptoms in the beginning but developed cyanoderma and increased MetHb levels close to 50% as time passed. This case highlights the need for all workers who handle methemoglobinemia-causing substances to be provided with access to a medical institution with plenty of experience in occupational and environmental medicine.

Ultimately, methemoglobinemia-causing substances, such as nitrobenzene and aniline, can be absorbed by workers at petrochemical plants in various ways, and symptoms may not appear for a few hours after exposure. Businesses that handle such substances must have a strict maintenance system in place, as well as a protection system for workers, including regular exposure check-ups and an emergency patient management system to ensure that all workers have access to timely diagnosis and treatment at an affiliated medical institution.

## Consent

Written informed consent was obtained from the patient’s guardian/parent/next of kin for the publication of this report and any accompanying images.

## Competing interests

We have no competing interests.

## Authors’ contributions

LCH and MJD conceived the study. CYH were involved in patient management. KSH and KDH collected medical and occupational information. LCH wrote the manuscript. JKH and MJD performed interpretation of the data and reviewed the manuscript. All authors read and approved the final manuscript.
